# Lower circulating platelet counts and antiplatelet therapy independently predict better outcomes in patients with head and neck squamous cell carcinoma

**DOI:** 10.1186/s13045-014-0065-5

**Published:** 2014-09-27

**Authors:** Saleh Rachidi, Kristin Wallace, Terry A Day, Anthony J Alberg, Zihai Li

**Affiliations:** Department of Microbiology and Immunology, 86 Jonathan Lucas, Suite 612, Charleston, SC 29425 USA; Hollings Cancer Center, 86 Jonathan Lucas, Suite 612, Charleston, SC 29425 USA; Department of Public Health Sciences, 68 President Street, BE 103, Charleston, SC 29425 USA; Head and Neck Tumor Center, Department of Otolaryngology-Head and Neck Surgery, Medical University of South Carolina, 171 Ashley Avenue, Charleston, SC 29425 USA

## Abstract

**Background:**

Head and neck squamous cell carcinoma (HNSCC) mortality rates have not shown significant reduction in decades. Platelets are being implicated in having cancer-promoting roles, an observation supported by the adverse outcomes associated with thrombocytosis in many malignancies associated with thrombocytosis. However, the prognostic significance of platelet counts in HNSCC is unknown. Here, we comprehensively investigate the predictive value of platelet counts at diagnosis and post-diagnosis antiplatelet treatment in the overall survival of HNSCC patients.

**Methods:**

The study population consists of 1051 pathologically confirmed HNSCC cases diagnosed between years 2000 and 2012 in a tertiary medical center. Platelet count was investigated as a predictor of survival by fitting Cox Proportional Hazards (CPH) regression models to generate Hazard Ratios (HR) and 95% confidence intervals (CI), while adjusting for age, sex, race, stage, treatment and smoking status. Finally, we evaluated the association between overall survival and antiplatelet medication intake after diagnosis.

**Results:**

Multivariable analysis showed an increased death rate in patients with thromobocytosis [HR 2.37, 95% CI 1.60-3.50)] and high normal platelet counts [HR 2.20, 95% CI 1.58-3.05] compared to the reference middle normal group. Post-diagnosis treatment with antiplatelet medications was inversely associated with death rate [HR 0.76, 95% CI 0.58-0.99].

**Conclusions:**

Higher platelet counts were associated with poorer prognosis in HNSCC patients, whereas antiplatelet agents were associated with better prognosis. Antiplatelet agents warrant evaluation in preclinical and clinical settings as a way to improve survival in HNSCC.

## Introduction

Head and neck cancer is common in many areas across the world and is mainly linked to tobacco smoking, alcohol consumption and Human Papilloma Virus (HPV) infections. Fifty-five thousand cases are newly diagnosed each year in the United States [1] and 550,000 worldwide [2]. Recent work has shown hematological parameters including neutrophils, lymphocytes and monocytes to predict outcome in head and neck cancers [3–5]. In addition, pretreatment thrombocytosis correlated with shorter survival in oral squamous cell carcinoma [6]. Intriguingly, thrombocytosis, which is highly prevalent in cancer patients [7], is a poor prognostic indicator in a number of cancers [8–13].

The relation between platelets and cancer progression suggests a possible role that extends beyond their hemostatic function [14,15]. More recently, platelets have been recognized as mediators of other regulatory functions in physiology such as angiogenesis, wound healing and immunomodulation [15]. Platelets secrete cytokines and growth factors such as TGF-β [16], VEGF [17], MMP-2, PF4 [18] and PDGF [19], which in turn induce hallmarks of cancer progression such as epithelial-mesenchymal transition (EMT) [20], angiogenesis, cell migration and/or proliferation. For example, PDGF induces the dimerization of PDGFR and EGFR, resulting in EGFR transactivation [21]. Platelet-derived TGF-β also acts on cancer cells to activate the Smad and NF-κB pathways, thus promoting cancer metastasis [22]. As a perpetuating mechanism, cancer cells can also produce soluble mediators such as IL-6 and GM-CSF, which stimulate thrombopoiesis [23,24]. Interestingly, the use of drugs with antiplatelet activity, such as aspirin, is associated with lower incidence and better prognosis in colon cancer and other cancers [25,26].

In the present investigation, we examine closely and systemically the relationship between platelet count in the peripheral venous blood and survival in head and neck squamous cell cancer (HNSCC), including oral, pharyngeal and laryngeal cancers. In particular, we investigate the prognostic significance of platelet counts within the normal and abnormal ranges to determine if there is a linear association between platelet number and clinical outcome. To further investigate the role of platelets in HNSCC, we studied the association of medications known to interfere with platelet function; anti-platelet medications and non-steroidal anti-inflammatory drugs (NSAIDs) [27], with survival of HNSCC patients.

## Subjects and methods

### Patient inclusion and exclusion criteria

The Cancer Registry at Hollings Cancer Center, Medical University of South Carolina (MUSC) was used to identify all cases of HNSCC (squamous cell cancers of the oral cavity, pharynx and larynx). The study population was comprised of histologically confirmed cases diagnosed between January 1, 2000 and June 30, 2012. After excluding patients diagnosed with a second primary cancer between January 1, 1993 (the earliest date recorded) and June 30, 2013, there were 1376 HNSCC patients available for the study. Patients without recorded platelet counts were also excluded, resulting in a total of 1051 patients available for analysis. The follow-up period ranged between 2 weeks and 156 months, with a median 25.7 months.

### Data collection

The MUSC Institutional Review Board approved all study activities. For each case, we abstracted data on demographic characteristics, clinical and pathological variables at diagnosis, treatment received and outcome using two different data sources: the Hollings Cancer Center (HCC) cancer registry and the MUSC Clinical Data Warehouse (CDW). The registry is part of a state mandated data system that collects cancer incidence on all cases in South Carolina. The CDW is a single, secure and integrated database extracted from the MUSC OACIS Clinical Data Repository, which includes patient demographics, ICD-coded diagnoses, ICD-coded procedures, medications and laboratory test results. These databases were subsequently linked, in a blinded fashion, through an honest broker at the CDW, and entered into a secured study database.

Independent variables obtained from the CDW included socio-demographic characteristics (age at diagnosis, sex and race); pre-treatment platelet count, lifestyle factors including smoking status (never, former and current) and alcohol use (never, former and current); and use of platelet-interfering medications, namely NSAIDs or antiplatelet medications, after diagnosis. NSAIDs included aspirin, celecoxib, rofecoxib, valdecoxib, diclofenac, etodolac, ibuprofen, indomethacin, ketorolac, meloxicam, nabumetone, naproxen, oxaprozin, sulindac and choline magnesium trisalicylate. Antiplatelet medications included cilostazol, clopidogrel, prasugrel, ticlopidine, abciximab, eptifibatide, tirofiban, anagrelide, dipyridamole, dipyridamole and aspirin. Intake of medications was based on the inpatient records of the CDW. Tumor-related variables obtained from the registry included tumor grade (well differentiated, moderately differentiated, poorly differentiated/undifferentiated); location of the primary tumor (oral cavity, pharynx, larynx); TNM stage (I, II, III, IV); and receipt of all first-line therapies (chemotherapy, surgery, radiation and/or other). Because there were only 5 cases with undifferentiated grade, we combined these with poorly differentiated grade into a single category for the purposes of analysis.

### Data analysis

The main study outcome was overall survival. Survival time was calculated as the time from diagnosis with HNSCC to death from any cause through July 19, 2013. Subjects alive as of this date were censored at the end of follow-up. Kaplan-Meier methods were used to generate median survival time and corresponding 95% confidence intervals for five classes of platelet count (low, low-normal, mid-normal, high-normal and high). Abnormal platelet count was defined as thrombocytopenia (“low”, <150,000/μL) or thrombocytosis (“high”, ≥400,000/μL). Within the normal range (150–399), we *a priori* sub-divided patients into approximate thirds [i.e. low-normal (150–229), mid-normal (230–314) and high-normal (315–399)]. Kaplan-Meier curves were plotted to graphically assess the relationship between the five categories of platelets (described above) and survival. The log-rank test was used to assess statistical significance.

We evaluated the association between the 5 platelet categories and survival by fitting univariate Cox Proportional Hazards (CPH) regression models. We also analyzed platelets as a continuous variable within the normal range. We chose to restrict the analysis to the normal range because the relationship between platelets and death was non-linear (u-shaped curve) in the lower tail of the distribution. To control for potential confounding variables, we performed univariate CPH for all other variables in Table 1 (i.e., age, sex, race, smoking status, alcohol use, treatment, tumor grade, location and clinical stage). Multivariable CPH models were then fit for the platelet variable and all other variables that were found to have a p-value of < 0.20 in the univariate analysis. Variables were retained in the final CPR model if they were significant at p < 0.05. Additionally, for each model, we examined two-way interactions between platelet measures and variables that were significant in the multivariable model. Interactions were retained in the final model if they were significant at p < 0.05.

**Table 1:** **Demographic and clinical characteristics within the five categories of platelet counts**

	**Platelet count categories**					
**Variable**	**Low (1–149) N = 67**	**Low normal (150–229) N = 361**	**Mid normal (230–314) N = 379**	**High normal (315–399) N = 169**	**High (≥400) N = 75**	**p-value**
**Age—yrs. Mean (SD)**	60.0 (10.7)	61.0 (10.8)	58.4 (11.9)	58.0 (10.8)	58.2 (11.5)	0.009
**Male—no. (%)**	55 (82)	302 (84)	273 (72)	117 (69)	53 (71)	<0.0001
**Race—no. (%)**						<0.0001
Caucasian	48 (72)	287 (79)	293 (77)	116 (69)	42 (56)	
African American	18 (27)	67 (19)	83 (22)	52 (31)	33 (44)	
Other	1 (1.5)	7 (2)	3 (1)	1 (0.5)	0 (0)	
**Smoker—no. (%)**						<0.0001
Never	6 (9)	63 (19)	67 (19)	18 (11)	4 (6)	
Former	20 (32)	125 (38)	111 (32)	40 (25)	14 (20)	
Current	37 (59)	145 (43)	173 (50)	102 (64)	52 (74)	
**Alcohol – no. (%)**						0.02
Never	17 (27)	99 (30)	108 (29)	36 (23)	7 (10)	
Former	13 (20)	55 (17)	54 (14)	26 (17)	9 (14)	
Current	34 (53)	174 (53)	177 (46)	94 (60)	50 (76)	
**Stage**						0.034
I	6 (11)	42 (15)	42 (14)	8 (7)	5 (8)	
II	9 (17)	37 (13)	35 (12)	10 (8)	4 (6)	
III	9 (17)	60 (21)	50 (17)	18 (15)	9 (14)	
IV	29 (55)	147 (51)	168 (57)	87 (71)	47 (72)	
**Grade**						0.90
Undetermined	20 (30)	106 (29)	103 (27)	42 (25)	16 (21)	
I. Well Differentiated	9 (13)	43 (12)	49 (13)	18 (11)	8 (11)	
II. Moderately	29 (43)	152 (42)	164 (43)	80 (47)	41 (55)	
III. Poorly or undifferentiated	9 (13)	59 (17)	63 (17)	29 (17)	10 (13)	
**Primary Site**						0.09
Oral	34 (51)	171 (47)	190 (50)	100 (59)	34 (45)	
Pharynx	13 (19)	101 (28)	107 (28)	42 (25)	18 (24)	
Larynx	20 (30)	89 (25)	82 (22)	27 (16)	23 (31)	
**Post-diagnosis NSAIDs and/or Anti-platelets**						0.33
No no. (%)	48 (72)	259 (72)	277 (73)	133 (79)	60 (80)	
Yes no. (%)	19 (28)	102 (28)	102 (27)	36 (21)	15 (20)	

All variables had complete data (n = 1051) except: smoking status (n = 977), alcohol use (n = 953), clinical stage (n = 822) and tumor grade, where the undetermined category accounted for n = 247 cases.

The methodology described above was used to examine the relationship between post-diagnosis use of NSAIDs/antiplatelets and survival. The association between any post-diagnosis NSAIDs/antiplatelet medication use (Yes or No) and survival was assessed while adjusting for age, race, smoking, treatment, tumor site, tumor grade, platelet level (continuous, or platelet classes) and clinical stage. We also examined the association of medication use and survival while excluding those with pre-diagnostic thrombocytopenia (who would not be expected to benefit from antiplatelet medications). The interaction between post-diagnostic NSAIDs/antiplatelet use and platelet classification survival was also evaluated.

## Results

### Relationship between platelet counts and clinical characteristics in HNSCC

Age inversely correlated with platelet counts, where patients in the low normal and low categories were slightly older than those in the other groups (Table 1). The majority of patients were males, but females were enriched in the higher platelet groups (mid-normal, high normal and high) (Table 1). In addition, African American patients were more represented in the thrombocytosis group where they comprised more than half of that population. Current smoking and alcohol consumption in turn were significantly associated with higher platelet counts (Table 1). Intriguingly, compared to those with stage I-III disease, higher platelet counts were observed in stage IV HNSCC (Table 1), although platelet levels did not significantly correlate with tumor grade or anatomic site (Table 1). No significant association was observed between intake of antiplatelet medications/NSAIDs and the platelet count. Finally, the proportion of deceased patients was higher in the high normal and thrombocytosis groups.

### Higher platelet counts are associated with worse overall survival

**Figure 1:**
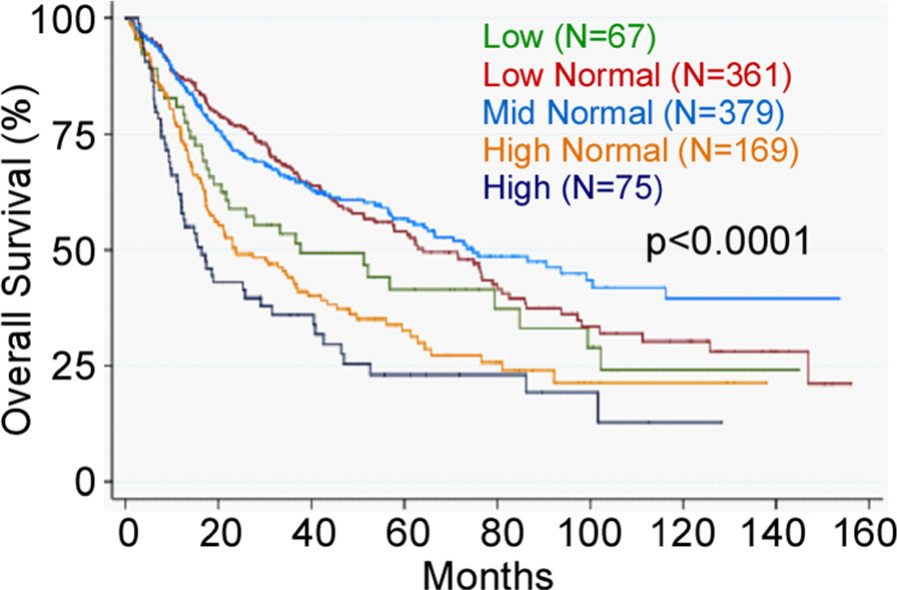
**High normal platelets and thrombocytosis are associated with worse overall survival.** (log-rank p < 0.0001).

Compared to those in the mid-normal category, patients in the high normal (adjusted HR 2.20; 95% CI 1.58-3.05) and thrombocytosis (adjusted HR 2.37; 95% CI 1.60-3.50) groups had more than two times higher death rates (Table 2 and Figure 1). The results for patients with platelet counts below the mid-normal category also trended toward poorer survival with adjusted hazard ratios of 1.25 (95% CI 0.93-1.68) in the low normal and 1.50 (95% CI 0.96-2.35) in the low categories, although these differences were not statistically significant.

**Table 2:** **Relative hazard of death in HNSCC patients based on platelet counts**

**Variables**	**Low (<150) N = 67**	**Low normal (150–229) N = 361**	**Mid-normal (230–314) N = 379**	**High normal (315–399) N = 169**	**HIGH (≥400) N = 75**
**Number dead (%)**	37 (55)	150 (42)	146 (39)	105 (62)	53 (71)
**Median Survival Months (95% CI)**	37.7 (20.7- 84.8)	64.0 (53.6-78.9)	74.7 (60.7 100.4)	23.8 (17.7 - 36.7)	16.3 (11.8 -29.2)
p-value for difference					<0.0001
**Overall Survival**					
Unadjusted HR (95% CI)	1.52 (1.06-2.18)	1.07 (0.85-1.34)	1.0 (reference)	1.98 (1.54-2.55)	2.64 (1.92 -3.61)
Adjusted HR (95% CI)	1.50 (0.96-2.35)	1.25 (0.93-1.68)	1.0 (reference)	2.20 (1.58-3.05)	2.37 (1.60-3.50)
**HR (95% CI) by Stage**					
I (n = 103)	0.41 (0.05-.3.31)	1.18 (0.33-4.20)	1.0 (reference)	1.60 (0.27-9.54)	0.35 (0.03-4.18)
II (n = 95)	1.99 (0.52-7.67)	1.40 (0.57-3.38)	1.0 (reference)	1.54 (0.48-4.99)	1.83 (0.45-7.40)
III (n = 146)	0.74 (0.16-3.40)	1.32 (0.62-2.82)	1.0 (reference)	2.43 (0.91-6.50)	1.86 (0.53-6.45)
IV (n = 478)	2.33 (1.34-4.10)	1.18 (0.78-1.78)	1.0 (reference)	2.50 (1.66-3.76)	2.47 (1.55-2.93)

Adjusted model controls for age, race, site, grade, treatment, smoking and stage.

P for interaction between stage and classes of platelets p = 0.31.

Strikingly, when platelet count was modeled as a continuous variable within the normal range, an increase of platelets by 100,000/μL resulted in a 32% increase in the hazard ratio (p = 0.001) in the unadjusted model. The increase in hazard was 30% (p = 0.01) after controlling for age, race, treatment, tumor site, tumor grade, smoking and stage.

The univariate association between platelet count and risk of death was assessed for three tumor sites (oral, pharynx, and larynx). Patients with thrombocytosis compared to those in the mid-normal range had a HR of 4.35 (95% CI 2.75-6.87) for oral cancer, 2.41 (95% CI 1.32-4.44) for pharyngeal cancer, and 1.48 (95% 0.78-2.80) for laryngeal cancer.

### Antiplatelet medications are associated with longer overall survival

**Figure 2:**
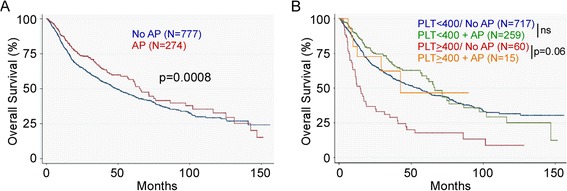
**Antiplatelets/NSAIDs are inversely associated with survival in HNSCC. A**. Intake of these medications confers better outcomes in the general HNSCC patient population, **B**. The benefit associated with intake of antiplatelets/NSAIDs is particularly observed in the thrombocytosis group, HR = 0.42, p = 0.06. AP: Antiplatelets. ns: Not significant.

Patients who were prescribed antiplatelet medications or NSAIDs after the date of diagnosis had a reduced risk of death compared to the other patients [HR 0.76, 95% CI 0.58- 0.99] after controlling for age, race, stage, grade, tumor site, treatment, smoking status, and platelet count (continuous) (Table 3 and Figure 2). The HR for medication use and death rate was 0.80 (95% CI 0.61-1.05) when we adjusted for age, race, stage, grade, tumor site, treatment, smoking status, and platelet classification. The association between the use of antiplatelet medications and death was stronger, however, when patients with thrombocytopenia were excluded: HR 0.73 (95 CI 0.55-0.98; p = 0.04). The association between medication intake and survival was assessed within each of the five platelet categories. Within the normal platelet range, intake of NSAIDs/antiplatelet conferred variable HRs in the protective direction, none of which was statistically significant. However, the protective association with intake of antiplatelet medications was strongest in the thrombocytosis group compared to those not taking medications [HR 0.42, 95% CI 0.17-1.05), p = 0.06] (Table 3).

**Table 3:** **Association of antiplatelet medications and risk of death**

	**HR 95% CI**		**p-value**
**Antiplatelet**	**No**	**Yes**	
Unadjusted	1.0	0.73 (0.58-0.92)	0.0008
Adjusted*	1.0	0.76 (0.58-0.99)	0.04
**Antiplatelet medications in 5 categories of platelet counts**			
Low	1.0	1.74 (0.74-4.09)	0.20
Low Normal	1.0	0.88 (0.55-1.42)	0.58
Mid Normal	1.0	0.68 (0.42-1.11)	0.12
High normal	1.0	0.87(0.48-1.51)	0.64
High	1.0	0.42 (0.17-1.05)	0.06

*Adjusted for age, race, tumor site, grade, treatment, platelet count and smoking.

## Discussion

The present study observed a clear association between higher platelet counts and worse overall survival in HNSCC. The results suggested that higher platelet levels, even in the normal range, are associated with poorer survival.

The evidence that the observed association between platelets and overall survival is genuine was solidified by the observation that the post-diagnostic intake of medications known to interfere with platelet function was associated with reduced risk of death in HNSCC patients, particularly among those with thrombocytosis. The fact that pharmacologic interventions aimed at lowering platelets were associated with prolonged survival provides quasi-experimental evidence that platelets are indeed linked with poor prognosis, reducing the likelihood that the observed association was due to bias or confounding factors. However, this evidence would be even stronger if it was clear that the observed association was due to an anti-platelet effect of these medications, but at the present time it cannot be ruled out that this association is attributable to the anti-inflammatory effects of many anti-platelet medications. However, given the recognized role of platelets in promoting cancer metastasis in preclinical animal models [22], it is likely that antiplatelet agents confer a beneficial effect and improve overall survival in cancer patients.

A limitation of our study is that we were unable to account for the amount and duration of intake of these agents, nor the start of their intake after diagnosis. The measurement error introduced likely resulted in bias toward the null hypothesis, but future studies with more detailed measurement of the anti-platelet medications are needed to refine our understanding on this issue.

Of note, our results suggested that low platelet counts (<150,000/μL) were associated with a trend towards worse overall survival compared to those in the low normal and mid-normal groups. However, the difference between the two groups did not reach statistical significance and is thus inconclusive. Alternatively, even if the null hypothesis is rejected by future studies with increased sample size, the possible explanations include pathological thrombocytopenia acting as a marker of poor hematological function associated with advanced disease. This consideration emphasizes the importance of considering a potential U-shaped relationship between platelet counts and cancer survival in future research on this topic.

## Conclusions

In conclusion, higher platelet counts were strongly associated with worse overall survival in a large cohort of head and neck cancer patients, whereas anti-platelet medications were associated with better survival. These findings, if replicated, have direct translational implications. Given the worse prognosis associated with higher platelet counts, and the potentially protective effect of platelet inhibitors in counteracting the effect of platelets, the investigation of anti-platelet agents in the treatment of HNSCC is warranted, especially that such agents are widely available and approved for other indications.
